# Supplementary material for: Testing vivo-morpholino mediated gene knockdown in threespine stickleback
Source: bioRxiv. 2026 Feb 24:2026.02.24.707669. Preprint. [Version 1] doi: 10.64898/2026.02.24.707669 (PMC13160006; doi:10.64898/2026.02.24.707669)
Supplement: Supplement 2 [file media-2.pptx]

## Slide 1
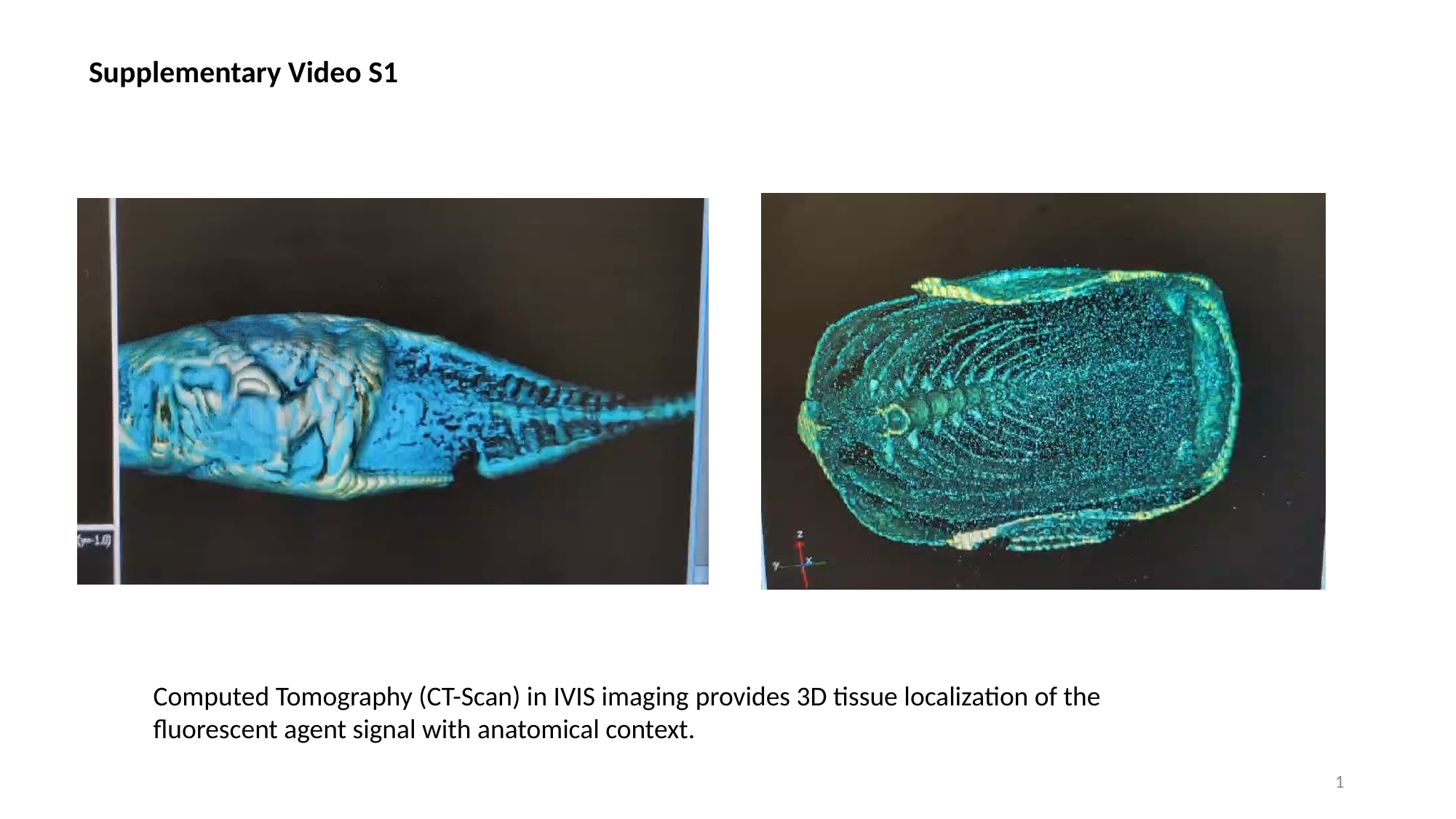

Supplementary Video S1
Computed Tomography (CT-Scan) in IVIS imaging provides 3D tissue localization of the fluorescent agent signal with anatomical context.
1
